# Utilization of a Novel Pathway in a Tertiary Pediatric Hospital to Meet the Sensory Needs of Acutely Ill Pediatric Patients

**DOI:** 10.3389/fped.2019.00367

**Published:** 2019-09-06

**Authors:** Neha Gupta, Chelsea Brown, Jennifer Deneke, Julian Maha, Michele Kong

**Affiliations:** ^1^Department of Pediatrics, University of Alabama at Birmingham, CPPI Suite, Birmingham, AL, United States; ^2^Children's of Alabama, Birmingham, AL, United States; ^3^KultureCity, Birmingham, AL, United States

**Keywords:** sensory processing difficulties, sensory sensitivities, sensory needs, sensory pathway, emergency department, children, acute illness

## Abstract

**Objective:** To identify pediatric patients with sensory sensitivities during a hospital visit, and to implement a clinical pathway that can meet their sensory needs. The goal is to remove barriers to care delivery that is related to the sensory need for pediatric patients who present with an acute medical illness.

**Methods:** The clinical pathway (identified as ‘Sensory Pathway’) was developed as a joint effort between key stakeholders within the community and medical providers. The pathway was conducted in a tertiary pediatric hospital from September 2016-April 2019. The main components of this pathway included- 1. Staff training; 2. Provision of sensory toolkits and story board; 3. Early collaboration with allied professionals; and 4. Early and continuous parental involvement. The Sensory Pathway was implemented first in the emergency department, followed by inpatient units. Patients triggered the pathway through caregiver or staff identification. Demographic of patients who triggered the pathway was extracted. A detailed qualitative analysis of any parents' feedback received was performed.

**Results:** A cohort of patients with sensory needs was identified amongst pediatric patients who presented to the hospital with an acute illness. The most common comorbidity associated with sensory sensitivity/need was Autism Spectrum Disorder (48%), followed by cerebral palsy (22.8%) and Attention-Deficit/Hyperactivity Disorder (16%). 1337 patients (51.8%) had a single comorbidity while 45.9% patients had more than one comorbidity. Only 1.3% patients had a known diagnosis of sensory processing disorder. The pathway was triggered in 2,580 patient visits with 1643 patients and 937 repeat visits. The vast majority of patients who triggered the pathway had a medical presenting complaint (vs. behavioral). The following themes emerged from the parents' feedback: 1. Additional help received specific to the child's sensory needs; 2. Feeling of comfort; and 3. Improved overall experience.

**Conclusion:** The Sensory Pathway identified a unique profile of pediatric patients who have sensory needs during their hospital stay. The pathway was successfully implemented for children with sensory need in our hospital across a wide range of demographic and with varied medical illness.

## Introduction

Patients with sensory processing difficulties or sensory sensitivities struggle with the integration of sensory inputs (either visual, auditory, tactile, taste, vestibular, and proprioceptive stimuli), often resulting in ineffective responses (1, 2). These challenges can be seen in children with comorbid conditions such as autism spectrum disorder (ASD) (3), cognitive disorder (4), prematurity (5), fetal alcohol syndrome (6), cerebral palsy (7), certain genetic conditions (for instance Trisomy 21 and Fragile X Syndrome) (8) and in patients with attention deficit hyperactivity disorder (ADHD) (9). Sensory processing or integration disorder can also exist independently in children without any known medical conditions (10).

When patients with sensory needs are acutely ill, the unknown hospital environment with varied sensory input can lead to an overwhelming experience. In addition to their heightened sensory sensitivities, these patients often have difficulties in communication (11). In a recent study, we found that both medical students and pediatric trainees had a perceived knowledge gap and discomfort when providing care to patients with ASD, particularly as it relates to sensory specific challenges (12). Pratt et al. similarly found that nurses indicated a lack of knowledge and discomfort when encountering patients with learning disabilities, with and without co-existing conditions such as ASD (13). Taken together, the patient's inherent difficulty with sensory processing, the environment and the providers' unfamiliarity with sensory related challenges can become a barrier to timely diagnosis of the presenting medical illness and the subsequent care delivery.

We developed this clinical pathway (identified as Sensory Pathway) to meet the needs of patients who might have sensory sensitivities when they present to the hospital with an acute illness. Our goal was to better identify who these patients were, and by education, use of specific toolkits, early collaboration with parents and allied professionals to facilitate medical care delivery and improve the overall patient experience for this cohort of patients.

## Materials and Methods

Via our partnership with KultureCity (www.kulturecity.org), a non-profit with a large network of special needs families, caregivers of children with known sensory processing disorder and sensory sensitivities were engaged prior to the design of this clinical pathway in order to better understand what their experiences were in the hospital settings, their biggest challenges, and what they deemed would be most helpful. Based on these family insights as well as existing knowledge of sensory processing disorder, the pathway was developed by a collaborative, interprofessional team consisting of physicians, nurses, occupational therapist, applied behavioral analyst, speech therapist and child live specialist. This pathway was implemented in a large tertiary care pediatric hospital from September 2016 to April 2019. The Sensory Pathway was first piloted in the pediatric emergency department (ED) at Children's of Alabama, Birmingham, Alabama in September 2016, followed by inpatient pediatric units in March 2018.

### Sensory Pathway

The Sensory Pathway included 4 main components: 1. Staff training; 2. Provision of sensory toolkits and storyboard; 3. Early collaboration with Child Life services; and 4. Early and continuous parental involvement. Staff training was provided by members of the sensory task force (pediatric intensivist, nurse educators and child life specialists) over several sessions to include all the nursing staff in the individual units (on average 4 to 6, 60-min sessions per unit), as well as to the Security Team at Children's of Alabama. Physicians including residents and faculty members were also exposed to the pathway during physician specific conferences. The training was focused on identification of a patient with sensory processing difficulties and use of various strategies and tools as needed. Training modules specifically included- 1. Understanding sensory processing challenges in special needs children, 2. Learning different methods of engagement and preventive strategies, 3. Learning effective communication strategies, 4. Learning environmental modifications, 5. Using sensory toolkits and storyboards, and 6. Learning de-escalation techniques during a sensory crisis. Refresher training was offered annually. After implementation of the Sensory Pathway, nursing “huddles” were performed daily to address questions and concerns specific to the unit.

Sensory toolkits were provided and made easily available for each unit. Components of the toolkits included items such as noise canceling headphones, fidget tools, light spinners and weighted lap pads (Appendix I). These tools were utilized based on the individual patient's sensory needs. For example, patients with auditory sensitivity were offered the noise canceling headphone, while those with tactile sensitivity were offered fidget tools of various shapes and textures. It is important to note that these items were selected based on patient preference, and often with the parents' input.

Storyboards were developed to help pre-condition a child to a particular procedure or encounter. These story boards describe the procedure or encounter in a precise and sequential way using simple and literal language. Examples include story boards for procedures such as laceration repair, intravenous line placement, urinary catheter insertion, or a trip to the radiology suite. An example of a storyboard (nasogastric tube placement) is shown in Appendix II.

A brief screener was used by providers to identify a patient who might have a sensory need (Appendix III). Patients triggered the Sensory Pathway through either caregiver or staff identification. It is important to note that any staff can trigger the pathway (even without the use of the screener) at any point of the hospital course based on the patients' clinical presentation or behavior. Once the Sensory Pathway was triggered, Child Life services was consulted. All patients on the pathway had access to different story boards used based on needs. Patients also had immediate access to the sensory toolkits, with items used based on the individual patient's preference, and with parental guidance. For instance, noise canceling headphones were offered to patients with auditory sensitivities. Patients were also strategically placed so that they were away from the busiest section of the individual unit. Throughout the entire process, providers were also encouraged to involve the parents early on, asking for parental input especially in regard to the child's preferences, known triggers and communication needs.

A Sensory Pathway survey was incorporated into the patient satisfaction surveys which are routinely performed hospital wide and made available to families. The surveys were voluntary, and any feedback received was reviewed by members of the sensory task force immediately to allow for modifications to the pathway if necessary. For instance, a theme that emerged in the ED was the need for a mobile sensory unit that could be used both as a calming and distraction tool for these sick children with a sensory need during procedures. Based on this feedback, a mobile sensory unit was made available in the ED for this specific need.

### Data Collection

All patients equal or >3 years of age who triggered the Sensory pathway in the ED or inpatient units were included in this study. A retrospective chart review was performed, and patients' demographic data was extracted from the electronic medical records including age, gender, race, location, chief presenting complaint, comorbidities and whether they were admitted or discharged (for those who alerted the pathway in the ED). Any sensory pathway related survey received by the hospital within this time frame was analyzed, and a quantitative analysis was performed for Questions 1–3 from the survey (Appendix III, Supplemental Table 1).

### Qualitative Analysis Methods

A detailed qualitative analysis of the comments and feedback given by these families in the patient satisfaction survey specific to Sensory Pathway was performed in order to determine the potential impact of the pathway on perceived patient experience. The comments from all the surveys were compiled for analysis. It is important to note that the surveys were submitted voluntarily by families, and not a requirement for participation in the Sensory Pathway.

Analysis was performed using NVivo 12 Pro (QSR International). First, all of the compiled comments were analyzed using a word frequency query. All the words with length >3 letters were included in the analysis. The most commonly used words by the families in the comments appeared in a report within the software. All similar words, which were stemmed from the same word, were sorted into synonym groups (e.g., words such as “help, helped, helpful, helping, and helps” counted toward the frequency of the word “help”). Based on the frequency, a weighted percentage of each word was calculated. A word loud was created based on word frequency. The size of the word located within the word cloud was determined by the frequency of the word(s) such that a more frequent word appeared larger than a less frequent word. Commonly used preposition or connector words, patient and staff names were excluded from the analysis.

The frequency of the words that were used to answer Question 4 (Appendix III, Supplemental Table 1) was reported (Appendix III, Supplemental Table 2) and analyzed to create a word cloud (Appendix III, Supplemental Figure 1).

### Ethics

This project was undertaken as a quality improvement (QI) project to systematically educate and implement current existing knowledge of sensory processing challenges, sensory overload and regulation in pediatric patients, and therefore was exempt from institutional ethical review. Because it was a QI project performed to improve care delivery and patient experience for children with sensory sensitivity, informed consent was not obtained from the families. The pathway was triggered based on provider recognition of need, or if the parents self-identified. Guidelines for reporting QI initiatives published by the SQUIRE Development Group were consulted for this manuscript (14). The Sensory Pathway survey was part of the patient satisfaction surveys which were performed routinely hospital wide. Parents were not interviewed for this survey, and completion of survey was voluntary as is routine for the hospital patient satisfaction survey.

## Results

The Sensory Pathway was triggered in 2,580 patient visits with 1,643 patients and 937 repeat visits. All 2,580 patient visits were included in this study. Table 1 outlines the demographic data of these patients including age gender, race, location at the time of sensory alert and disposition (whether admitted or discharged for those who alerted the pathway in the ED). Approximately 90% of the patients triggered the pathway in the ED.

**Table 1:** Demographics of patients identified with a sensory need.

**Characteristics**	***n***	**%**
**AGE**		
3–6 years	705	27.3
6–10 years	753	29.2
10–14 years	537	20.8
14–18 years	395	15.3
≥18 years	190	7.4
**GENDER**		
Female	755	29.3
Male	1,825	70.7
**RACE**		
White	1,680	65.1
Black	867	33.6
Asian	22	0.85
Hispanic	4	0.15
Other	7	0.3
**PATIENT LOCATION AT THE TIME OF TRIGGER**		
Emergency department	2,335	90.5
Discharged	1,603	68.7
Admitted	732	31.3
Inpatient	245	9.5

**Figure 1:**
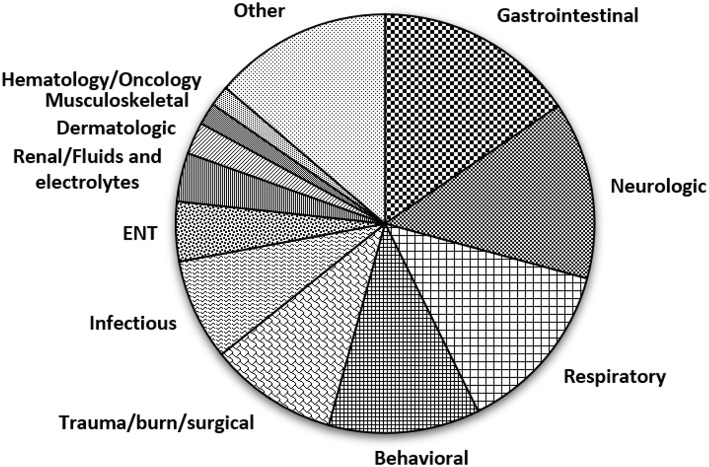
Pie chart showing the chief presenting complaints of patients who triggered the Sensory Pathway. Footnote- Gastrointestinal complaints include malnutrition, enteritis, esophagitis, acid reflux, gastric ulcer, gastritis, gastroparesis, inflammatory bowel disease, appendicitis, constipation, gastrointestinal hemorrhage, vomiting abdominal pain, foreign body, liver transplant and problem with colostomy or gastrostomy; Neurologic complaints include seizures, postconcussion syndrome, extrapyramidal movement disorder, myoclonus, migraine, cerebral ischemia, insomnia, hemiplegia, quadriplegia, hydrocephalus, pain, encephalopathy, gait and movement disorders, altered mental status, amnesia, headache and syncope; Respiratory complaints include cystic fibrosis, obstructive sleep apnea, tracheitis, croup, upper respiratory infection, pneumonia, cough, dyspnea, stridor, respiratory failure, wheezing, cyanosis and chest pain; Behavioral complaints include hallucinations, impulsiveness, suicidal ideation and physical violence; Trauma/burn/surgical complaints include burns, motor vehicle accident, bowel obstruction, injuries and fractures, hernia, abrasions, contusions and laceration; Infectious complaints include urinary tract infections, sepsis, viral and fungal infections; ENT complaints include otitis media, pharyngitis and foreign body, Renal/Fluids and electrolytes complaints include dehydration, acidosis, glomerulonephritis and electrolyte disturbance; Dermatologic complaints included skin infections and skin rash; Musculoskeletal complaints include bone and muscular pain, joint dislocation, hemarthrosis, scoliosis, dorsalgia, congenital deformities and pathological fractures; Hematology/Oncology complaints include anemia, pancytopenia, purpura, neutropenia, leukemia, lymphoma and solid tumors; Other complaints include genital problems, ingestions and poisoning, endocrine, cardiovascular and ophthalmologic complaints, dental and oral problems and problems related to devices like tubes and shunts.

The median age of the population was 8.8 years (interquartile range, 5.75–13.5 years). Overall, the primary presenting complaint was medical in nature, with only <12% presenting with a behavioral complaint. Of the medical complaints, the most common presenting complaints were gastrointestinal problems (15.5%), neurologic problems (13.7%) and respiratory problems (13.4%), while the least common were endocrine problems (0.8%), ophthalmologic problems (0.7%) and cardiovascular problems (0.6%) (Figure 1).

**Figure 2:**
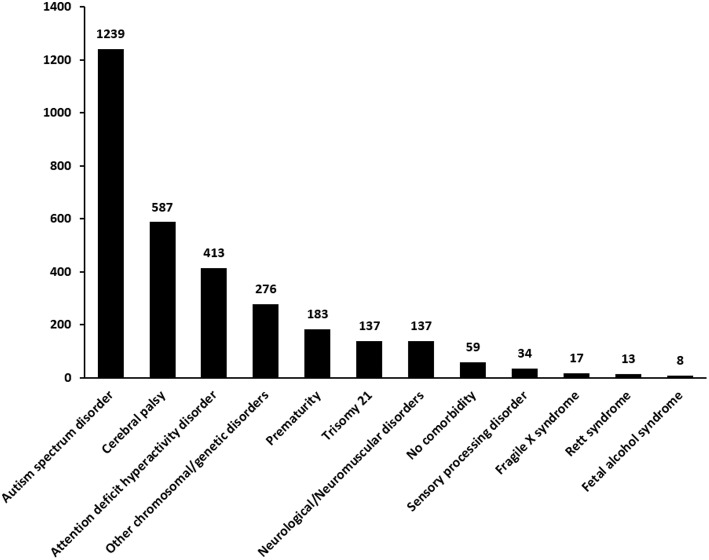
Comorbidities seen in patients with a sensory need who triggered the Sensory Pathway.

One thousand three hundred thirty-seven patients (51.8%) had a single comorbidity while 45.9% patients had more than one comorbidity. The most common comorbidity associated with sensory sensitivity was ASD (48%), followed by cerebral palsy (22.8%) and ADHD (16%) (Figure 2). Only 34 (1.3%) patients had a known diagnosis of sensory processing disorder on arrival to the hospital. 2.3% patients did not have any associated comorbidity.

100% of the families who responded to Q2 of the survey (“Do you feel your child has improved care and treatment related to the use of the sensory alert pathway?” Appendix III, Supplemental Table 1) reported that their child had improved care and treatment with the use of Sensory Pathway. The most beneficial part of the pathway was reported to be the supplies/tools (87%) followed by staff approach (65.2%), triage (26.1%) and storyboard (17.4%) (Appendix III, Supplemental Table 1).

A few direct quotes from the families are listed below.

“Having an autistic child is challenging but coming to the ED is even more of a challenge. But having the weighted blankets…sensory box and especially the Vecta…have greatly improved our stay. Over the last few months the sensory friendly changes to the ED have really made a difference for us. We are very thankful for the staff making the efforts to assist with special needs sensory sensitive patients…”

“We spend a lot of time at Children's and the changes with the sensory pathway have made such a difference. It helps with getting x-rays and has decreased the number of meltdowns. The Vecta is one of my son's favorite items.”

“I love that the plan was not immediate sedation, even though my child is extremely difficult. Toys are extremely beneficial for hospital visits, especially difficult ER visits. Thank you for your patience with us.”

### Qualitative Analysis Results

For the qualitative analysis, a word cloud was formed from the parents' feedback (Appendix III, Supplemental Figure 1). Themes identified included (1) Additional help received specific to the child's sensory needs; (2) Feeling of comfort and care; and (3) Improved overall experience.

## Discussion

Abnormal sensory behaviors are highly prevalent in children with ASD. However, sensory symptoms such as hyperresponsiveness, hyporesponsiveness, and sensory seeking behaviors have also been observed in children with other developmental disorders and even in those with normal development (15, 16). Studies have reported that the prevalence of sensory processing disorder can be as high as 50% in patients without disabilities (10) and up to 90% in those with disabilities such as ASD, ADHD and cognitive disorders (17). In our patient population, over a period of 32 months, we found that the majority of the patients who triggered the Sensory Pathway had multiple comorbidities including ASD, ADHD, cerebral palsy, prematurity and chromosomal disorders. The most common diagnosis was ASD, followed by cerebral palsy and ADHD (Figure 2). 90% of the patients triggered the pathway in the ED. This is a direct reflection of where the pathway was first initiated (ED), followed by extension to inpatient units. Furthermore, patients who triggered the pathway in the ED will continue to be on the pathway until the time of discharge. Inpatient unit triggers only captured patients who were admitted directly to the units and those who were admitted from the ED but did not trigger the pathway there.

In a study by Cohen-Silver et al., the majority of patients with ASD presented to the ED with a medical complaint rather than a behavioral problem (18). Most patients with sensory sensitivities have a co-existing condition, and often these patients have a higher rate of chronic health issues such as gastrointestinal problems compared to their peers (19). It is increasingly recognized that for many of these patients, their unique inherent core characteristics may impact the management of their presenting medical illness (20–22). In our patient population, 88.3% of the patients presented with non-behavioral complaints, highlighting the importance for all frontline providers to have an awareness of challenges related to sensory sensitivities, and ways to prevent or mitigate them.

Recently, Muskat et al. identified a gap between the needs of patients with ASD and the care provided to them in the hospital (20). In that study, many parents reported a negative hospital experience, ineffective communication, and poor recognition of their child's sensory need by the providers (20). In another study, sensory issues were also reported in non-autistic patients with and without disabilities and perceived to impede medical care (23). The hospital environment, especially the ED, can be overwhelming for a patient with sensory processing difficulties. Often the environment is intense, resulting in numerous and varied sensory exposure to the patient (24), including bright and harsh lights, loud noises, and intense smell. The wait time can be long, and there are often many physical interactions between the provider and patients, as well as the need for procedures. The hospital environment can easily trigger a sensory overload that can manifest as behavioral outburst and exacerbate the difficulties in making timely diagnosis and providing medical care. With the Sensory Pathway, we trained providers to be able to recognize sensory issues and provided them with both the knowledge and skill set to manage the sensory environment. Once identified, patients are placed in a quieter location, and given access to sensory toolkits and story boards (if needed) in order to minimize their sensory stimulation. Patients could be identified at any point during their hospital course, by any provider, allowing for a broad capture of patients in order not to miss any patients with a potential sensory need. In addition, triggering of the Sensory Pathway was not limited to patients with a diagnosis of a genetic condition or sensory processing disorder, but also by patients with sensory sensitivities as reported by caregivers. This allowed for all patients with a sensory need, including those who may have developed it while hospitalized to be identified. Education of all frontline providers was key as it allowed for early identification, and for the providers to be equipped with the knowledge, skill set and additional resources to help mitigate barriers posed by the sensory sensitivities.

Effective communication is also key in providing optimal care to special needs children, many of whom are non-verbal (20, 25). With the Sensory Pathway, providers are trained to ask about preferred method of communication, whether it is verbal communication, with augmentative device or with visual cue cards. By identifying the patient's preferred method of communication, information exchange is optimized, allowing for better care delivery. Providers are also trained to use concrete or literal language that can be less confusing, particularly when a patient is having a sensory overload. In a situation whereby there are multiple providers, it is recommended that one person takes the lead in the communication with the patient at any one time. Providers are also encouraged to pay closer attention to non-verbal cues, and to allow patients more time to respond.

In the Sensory Pathway, sensory toolkits (headphones, fidget tools, weighted lap pads, etc.) are made available to the patients, and used according to their preference and with parental input. Although families often have similar sensory items at home for comfort or distraction, due to the sudden and unexpected nature of the hospital visit, these items are often not brought with the child. By having these sensory toolkits as a resource on the Sensory Pathway, they can be readily available, and used both as a comfort and distraction item. In addition, story boards are utilized to pre-condition patients to certain procedures such as nasogastric tube placement, urinary catheter placement, laceration repair, etc. Other studies have shown that these types of narrative can be used successfully to reduce anxiety and can be effective in decreasing inappropriate behaviors (11, 26, 27).

In a recent meta-synthesis on parents' perspectives of children with ASD, parents reported frustration when their concerns were dismissed by providers and felt helpless when they were not regarded as experts for their child (28). Family members of children with disabilities are valuable resources in the assessment of their child's unique needs and are often the “translators” for those who are minimally verbal (29–31). Parental involvement early on and throughout the hospital course is essential and can improve patient care delivery and experience (29, 32). By being an active participant in the care of their children, parents not only help create a more comfortable environment for their children, they can also alleviate provider anxiety, which together can facilitate a child's medical care (32). With the Sensory Pathway, parents are intimately involved from the onset, and their unique knowledge of their child used to identify preferences, triggers, baseline behaviors, and communication strategies. In addition, another important component of the Sensory Pathway is the early involvement of allied professionals, in particular the Child Life Specialist once the pathway is triggered. The Child Life Specialist can provide additional resources to the medical staff, as well as provide support to the patients and their family during the hospital stay.

With the implementation of the Sensory Pathway, the main trend that emerged from the parents' feedback was having the individual unique sensory needs of their children met, and the perceived improved overall experience. This is important as improved parental satisfaction with health care of children with disabilities has shown to decrease anxiety and improved well-being and quality of life (33).

The Sensory Pathway has several limitations. Our findings reflect the experiences of a single academic center, and clinical outcome measures such as duration of wait time, length of stay, use of sedations etc were not captured. Quantitative measures of comfort/delirium/agitation were also not captured and may have been useful in quantifying the effect of the interventions performed via the Sensory Pathway. The rate of adoption or triggering of the pathway is unknown. We were however able to identify a unique cohort of patients with sensory needs, most of whom presented with a medical complaint (vs. behavioral). Also, due to the voluntary nature of the sensory pathway survey, the response rate of the survey was low. Most patients who utilized the pathway verbalized their satisfaction which was not officially documented. There is also a possibility that non-responders may not have liked the Sensory Pathway, and therefore decreased the positive response received (100% of responders reported improved care). We were, however, able to use the survey as a platform for parents to express specific concerns (Q4: open comment section), and to use the information to identify areas of improvement. There is also the possibility that over time implementation efforts may dissipate. We have attempted to address this need by identifying local unit champions and stakeholders, by performing frequent “check ins,” unit “huddles” and by having refresher trainings. Our intent is to use information gleaned from this pathway for larger scale multicenter implementation, and to inform how we provide best care. It is also important to note that no additional staff was hired for implementation of this pathway- our aim was to empower all frontline staff with the knowledge and skillset necessary to mitigate some of the barriers to care delivery that was related to the sensory needs.

In conclusion, we were able to identify a cohort of patients with sensory needs, many of whom have comorbidities, and presented with a medical complaint. The Sensory Pathway was developed to meet these sensory barriers when these patients presented to the ED or inpatient units with an acute illness. We were able to successfully implement the pathway in a large tertiary children's hospital, with utilization across a wide range of patient demographic, and with varied medical illness. Our goal is to extend this program to other inpatient and outpatient units and validate our findings on a large scale. We intend to use our findings to offer relevant and useful data for clinicians, health researchers and policy makers in order to improve medical care delivery and patient experience in this patient cohort. There needs to be ongoing collaboration between health care administrators, health care providers and families to inform the process of ensuring that goals of care are met for this growing population.

## Data Availability

All datasets generated for this study are included in the manuscript/Supplementary Files.

## Ethics Statement

Ethical review and approval was not required for the study on human participants in accordance with the local legislation and institutional requirements. Written informed consent from the participants' legal guardian/next of kin was not required to participate in this study in accordance with the national legislation and the institutional requirements.

## Author Contributions

All authors listed have made a substantial, direct and intellectual contribution to the work, and approved it for publication.

### Conflict of Interest Statement

JM and MK are founders of KultureCity. The authors declare that the research was conducted in the absence of any commercial or financial relationships that could be construed as a potential conflict of interest.
