# Supplementary material for: Utilization of a Novel Pathway in a Tertiary Pediatric Hospital to Meet the Sensory Needs of Acutely Ill Pediatric Patients
Source: Front Pediatr. 2019 Sep 6;7:367. doi: 10.3389/fped.2019.00367 (PMC6742947; doi:10.3389/fped.2019.00367)

Appendix I: Example of a sensory toolkit checklist

TACTILE/TOUCH

Fidget tools: 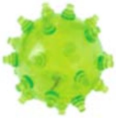 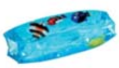 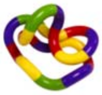

OLFACTORY/SMELL

Scented tools: 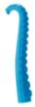 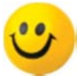

AUDITORY/HEARING

Sound maker: 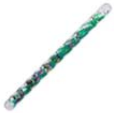

Headphone: 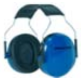

VISUAL/VISION

Tools with lights/motion: 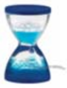 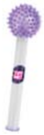 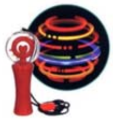

Sunglasses: 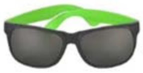

PROPRIOCEPTIVE/VESTIBULAR

Mini Massager: 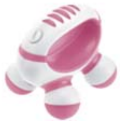

Weighted Blanket: 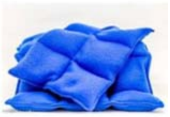

Appendix II: Example of a storyboard: Nasogastric (NG) tube placement

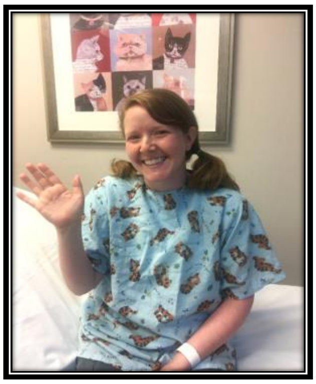

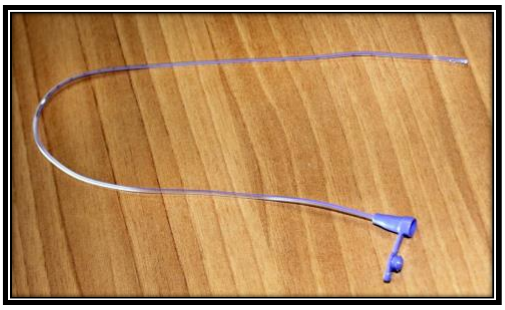

I am getting an NG tube.
An NG tube is a small bendy tube that will go into my nose and down to my stomach.

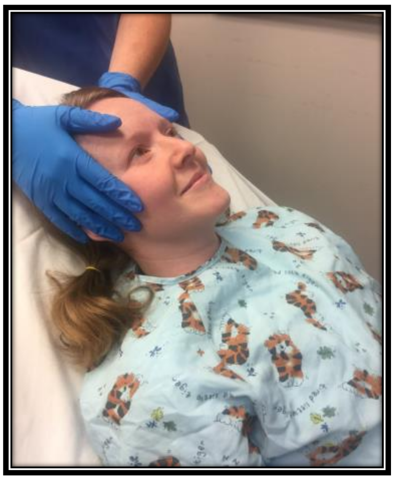

For my NG tube I can choose to sit in someone's lap or by myself.

My big job is to keep my head and body very still while I get my NG tube.
If I have a hard time staying still by myself someone can help hold my head to remind me to stay still.

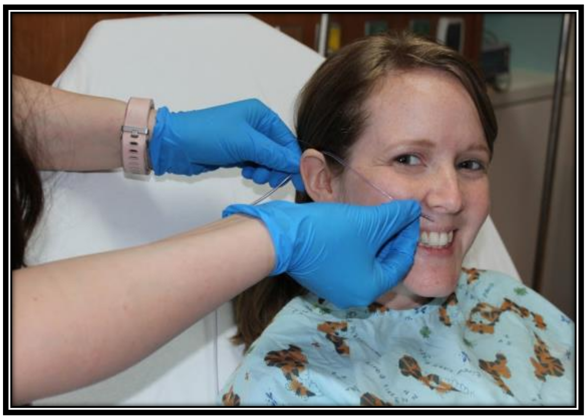

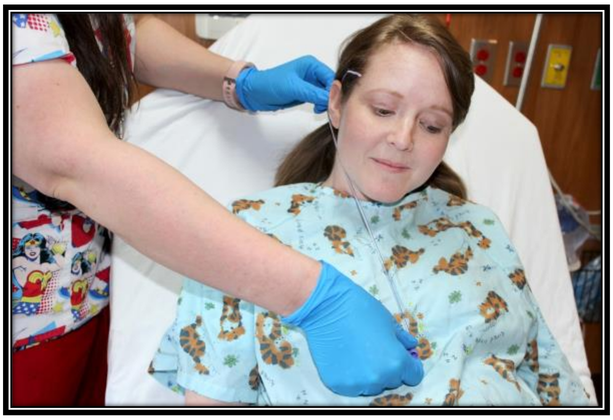

First the nurse will measure from my
nose to my ear using the bendy tube.

The nurse will then measure from my ear to my sternum.

My sternum is below my chest and above my belly button.

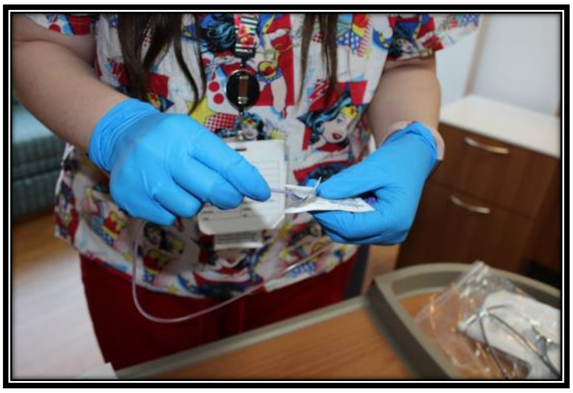

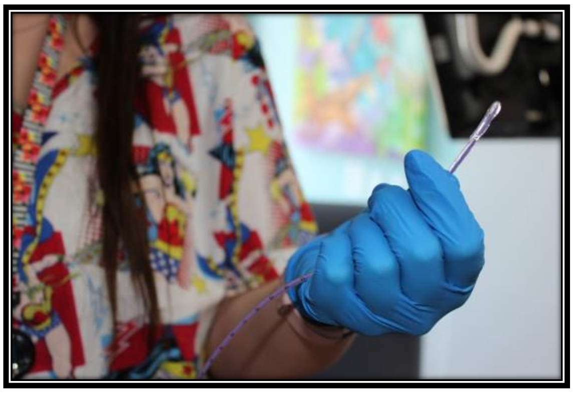

The nurse will then put a clear jelly on the end of the bendy tube.
This gel helps the tube slide into my nose easier.

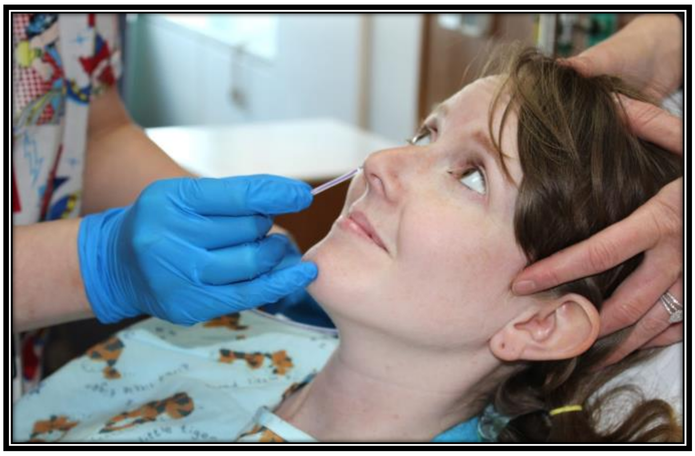

I am now ready for my NG tube.

The nurse will start to slide the tube into my nose.
This may tickle my nose and throat.
The tickle may make cough.
That's ok! I can help the tube go to the right place faster by swallowing like when I drink my favorite drink.

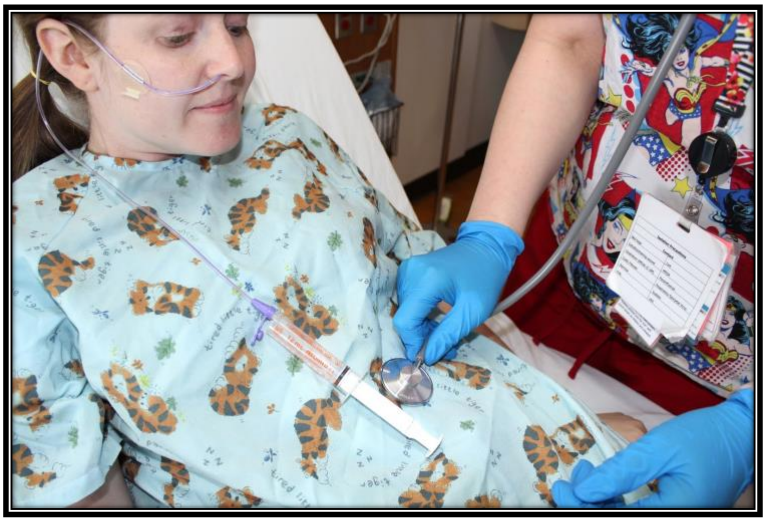

Once the tube is in the nurse will use a stethoscope to listen to my stomach.
This helps the nurse know that the tube is in the right place.

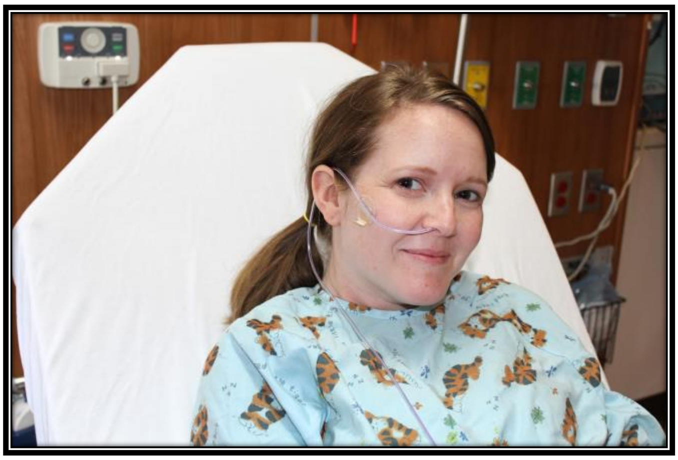

The nurse will then use tape and band aids to keep the tube in place until I do not need it anymore.

I am going to do great!

Appendix III

Brief Screener for Sensory Pathway

Patient **Date**

Reported by **Relation to patient**

Parents/Guardians can provide valuable information about their child's ability to cope with healthcare visits.

Staff: Please obtain these answers by asking the parent/guardian or by giving them this form to complete.

Parent/Guardian: Please answer the following questions as well as you can. Your responses can help us assess your child's support needs.

Sensory Coping

- Does your child usually get upset or overwhelmed with loud noises, bright lights, large crowds or other kinds of sensory overload?

  Yes ☐ **Depends** ☐ **No** ☐

- If you selected "depends," are aspects of this visit likely to be challenging for your child?

Anxiety and Coping

- Does your child usually get upset or anxious during visits to the doctor or hospital?

  Yes ☐ **Depends on the type of visit** ☐ **No** ☐

- If you selected "depends," are aspects of this visit likely to be challenging for your child?

Temperament

- Does your child react negatively to changes or need a lot of time before settling into situations?

  Yes ☐ **Depends on the type of situation** ☐ **No** ☐

- If you selected "depends," are aspects of this visit likely to be challenging for your child?

Respond

- If you answered yes to any of the questions above, what are some of the things that helps calm your child?

Staff use only:

Staff Name: **Unit/Area:**

Support Need: Low ☐ High ☐

Check "High" if any Yes is checked, and facilitate appropriate support.

Supplemental Table 1. Sensory Pathway survey questions with caregiver responses

Survey Questions	Response	n (%)
Q1. Has your child been to Children's of Alabama Emergency Room before?	Yes<br>No<br>Unanswered	20 (87)<br>2 (8.7)<br>1 (4.3)
Q2. Do you feel your child has improved care and treatment related to the use of the sensory alert pathway?	Yes<br>No<br>Unanswered	22 (95.7)<br>0 (0)<br>1 (4.3)
Q3. What part of the pathway do you believe was most beneficial for your child?<br>(You can select more than one answer)	Triage<br>Storyboard<br>Approach by hospital staff<br>Supplies/tools	6 (26.1)<br>4 (17.4)<br>15 (65.2)<br>20 (87)
Q4. Additional comments	Please see Table 2 and Figure 1 for word frequency analysis	

Supplemental Table 2. Word frequency analysis for caregiver comments

Word	Length	Count	Weighted Percentage	Similar Words
help	4	13	4.28%	help, helped, helpful, helping, helps
sensory	7	11	3.62%	sensory
needs	5	8	2.63%	need, needs
son	3	6	1.97%	son, sons
thank	5	6	1.97%	thank, thankful
toys	4	5	1.64%	toys
great	5	5	1.64%	great, greatly
child	5	4	1.32%	child
children	8	4	1.32%	children
especially	10	4	1.32%	especially
issues	6	4	1.32%	issues
really	6	4	1.32%	really
vecta	5	4	1.32%	vecta
visit	5	4	1.32%	visit, visits
time	4	4	1.32%	time, times
love	4	3	0.99%	love, loved
pathway	7	3	0.99%	pathway, pathways
use	3	3	0.99%	use, used
welcomed	8	3	0.99%	welcome, welcomed

changes	7	3	0.99%	changes
feel	4	3	0.99%	feel
last	4	3	0.99%	last
lot	3	3	0.99%	lot
much	4	3	0.99%	much
special	7	3	0.99%	special
staff	5	3	0.99%	staff
amazing	7	2	0.66%	amazing
appreciate	10	2	0.66%	appreciate, appreciated
autistic	8	2	0.66%	autistic
best	4	2	0.66%	best
blankets	8	2	0.66%	blankets
calm	4	2	0.66%	calm
challenge	9	2	0.66%	challenge, challenging
comes	5	2	0.66%	comes, coming
comfort	7	2	0.66%	comfort, comfortable
difference	10	2	0.66%	difference
difficult	9	2	0.66%	difficult
entertained	11	2	0.66%	entertained
everyone	8	2	0.66%	everyone
extremely	9	2	0.66%	extremely
friendly	8	2	0.66%	friendly
good	4	2	0.66%	good
hospital	8	2	0.66%	hospital
immediate	9	2	0.66%	immediate, immediately
items	5	2	0.66%	items
kept	4	2	0.66%	kept
kids	4	2	0.66%	kids
light	5	2	0.66%	light, lights
many	4	2	0.66%	many
program	7	2	0.66%	program
relax	5	2	0.66%	relax, relaxed
stay	4	2	0.66%	stay
think	5	2	0.66%	think
tools	5	2	0.66%	tools

trip	4	2	0.66%	trip
weighted	8	2	0.66%	weighted
wonderful	9	2	0.66%	wonderful
accommodating	13	1	0.33%	accommodating
acted	5	1	0.33%	acted
add	3	1	0.33%	add
adding	6	1	0.33%	adding
alert	5	1	0.33%	alert
ambulance	9	1	0.33%	ambulance
anxiety	7	1	0.33%	anxiety
arrive	6	1	0.33%	arrive
assist	6	1	0.33%	assist
attention	9	1	0.33%	attention
awesome	7	1	0.33%	awesome
babies	6	1	0.33%	babies
beneficial	10	1	0.33%	beneficial
box	3	1	0.33%	box
coa	3	1	0.33%	coa
continue	8	1	0.33%	continue
create	6	1	0.33%	create
decreased	9	1	0.33%	decreased
diligently	10	1	0.33%	diligently
distract	8	1	0.33%	distract
dream	5	1	0.33%	dream
ease	4	1	0.33%	ease
efforts	7	1	0.33%	efforts
emergency	9	1	0.33%	emergency
enjoyed	7	1	0.33%	enjoyed
environment	11	1	0.33%	environment
everything	10	1	0.33%	everything
excellent	9	1	0.33%	excellent
experiences	11	1	0.33%	experiences
extra	5	1	0.33%	extra
fact	4	1	0.33%	fact
fast	4	1	0.33%	fast

favorite	8	1	0.33%	favorite
felt	4	1	0.33%	felt
full	4	1	0.33%	full
getting	7	1	0.33%	getting
give	4	1	0.33%	give
happy	5	1	0.33%	happy
home	4	1	0.33%	home
hours	5	1	0.33%	hours
huge	4	1	0.33%	huge
idea	4	1	0.33%	idea
improved	8	1	0.33%	improved
introduced	10	1	0.33%	introduced
investment	10	1	0.33%	investment
ipad	4	1	0.33%	ipad
know	4	1	0.33%	know
labs	4	1	0.33%	labs
length	6	1	0.33%	length
likes	5	1	0.33%	likes
little	6	1	0.33%	little
live	4	1	0.33%	live
locks	5	1	0.33%	locks
machine	7	1	0.33%	machine
malfunction	11	1	0.33%	malfunction
man	3	1	0.33%	man
meltdowns	9	1	0.33%	meltdowns
mirrors	7	1	0.33%	mirrors
months	6	1	0.33%	months
mood	4	1	0.33%	mood
music	5	1	0.33%	music
never	5	1	0.33%	never
nice	4	1	0.33%	nice
number	6	1	0.33%	number
nurse	5	1	0.33%	nurse
older	5	1	0.33%	older
one	3	1	0.33%	one

optic	5	1	0.33%	optic
overall	7	1	0.33%	overall
patience	8	1	0.33%	patience
patients	8	1	0.33%	patients
plan	4	1	0.33%	plan
please	6	1	0.33%	please
problems	8	1	0.33%	problems
process	7	1	0.33%	process
provided	8	1	0.33%	provided
puzzles	7	1	0.33%	puzzles
rays	4	1	0.33%	rays
reassurance	11	1	0.33%	reassurance
recognized	10	1	0.33%	recognized
room	4	1	0.33%	room
safe	4	1	0.33%	safe
sedation	8	1	0.33%	sedation
see	3	1	0.33%	see
seizures	8	1	0.33%	seizures
sensitive	9	1	0.33%	sensitive
service	7	1	0.33%	service
skip	4	1	0.33%	skip
smiles	6	1	0.33%	smiles
sooner	6	1	0.33%	sooner
spectrum	8	1	0.33%	spectrum
spend	5	1	0.33%	spend
spinning	8	1	0.33%	spinning
suggestion	10	1	0.33%	suggestion
super	5	1	0.33%	super
tall	4	1	0.33%	tall
tell	4	1	0.33%	tell
things	6	1	0.33%	things
though	6	1	0.33%	though
today	5	1	0.33%	today
tonight	7	1	0.33%	tonight
took	4	1	0.33%	took

triage	6	1	0.33%	triage
type	4	1	0.33%	type
upset	5	1	0.33%	upset
vision	6	1	0.33%	vision
waiting	7	1	0.33%	waiting
way	3	1	0.33%	way
wish	4	1	0.33%	wish
worked	6	1	0.33%	worked
years	5	1	0.33%	years

Supplemental Figure 1. Word cloud representing the caregiver comments

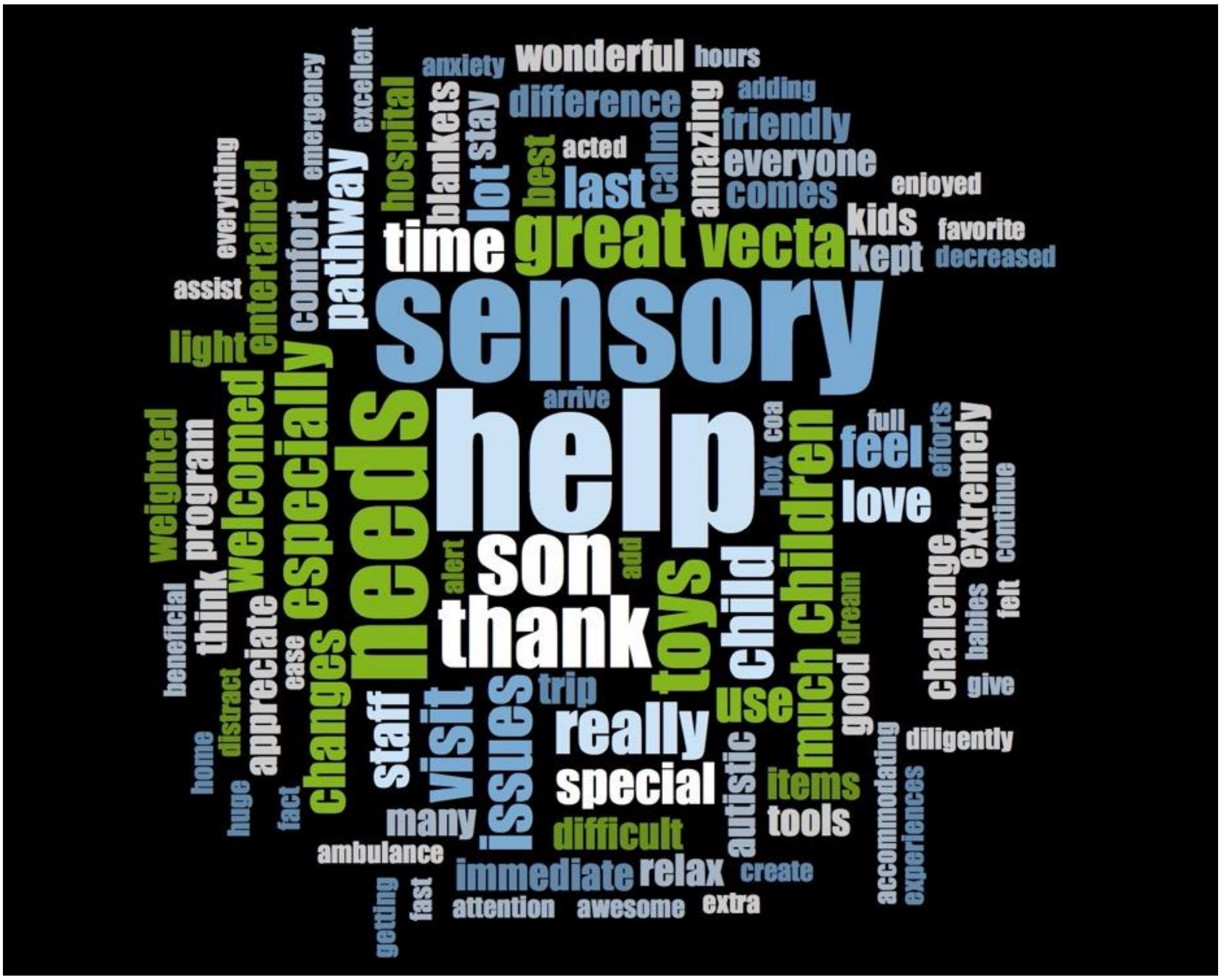

Supplement: Supplementary file 1 [file Data_Sheet_1.pdf]
